# Self-reported habitual snoring and health-related quality of life among adults attending community health centers in Shanghai, China: a cross-sectional study

**DOI:** 10.3389/fpubh.2026.1891156

**Published:** 2026-08-24

**Authors:** Xin Ge, Yuhui Sheng, Ruijie Gong, Suping Wang, Shangbin Liu, Chen Xu, Yuejin Yu, Yuan Yao, Yong Cai, Zhongqing Xu

**Affiliations:** 1 Institute of Clinical Research, Tongren Hospital, Shanghai Jiao Tong University School of Medicine, Shanghai, China; 2 Department of Dermatology, Longhua Hospital, Shanghai University of Traditional Chinese Medicine, Shanghai, China; 3 Shanghai Xuhui Center for Disease Prevention and Control (Shanghai Xuhui Health Inspection Agency), Shanghai, China; 4 Shanghai Jiao Tong University School of Medicine, Shanghai, China; 5 Institute of Medical Education, China Academy of Hospital Development, Shanghai Jiao Tong University, Shanghai, China; 6 Department of Otorhinolaryngology, Seventh People's Hospital of Shanghai University of Traditional Chinese Medicine, Shanghai, China; 7 Department of Gerontology, Tongren Hospital, Shanghai Jiao Tong University School of Medicine, Shanghai, China; 8 Institute of Community Medicine, China Academy of Hospital Development, Shanghai Jiao Tong University, Shanghai, China; 9 Department of General Practice, Tongren Hospital, Shanghai Jiao Tong University School of Medicine, Shanghai, China

**Keywords:** community health centers, habitual snoring, health-related quality of life, primary care, SF-12

## Abstract

**Background:**

Habitual snoring is a common symptom in primary care but is often overlooked as a minor nuisance. Although sleep-disordered breathing has been associated with adverse health outcomes, the association between self-reported habitual snoring and health-related quality of life (HRQoL) remains insufficiently characterized among adults attending community health centers in China. This study examined the association of habitual snoring with physical and mental HRQoL in this setting.

**Methods:**

We conducted a cross-sectional study among 2,312 adults recruited from community health centers in Shanghai, China. Habitual snoring was defined as self-reported snoring on at least 3 nights per week. HRQoL was assessed using the 12-item Short Form Health Survey (SF-12), from which the Physical Component Summary (PCS) and Mental Component Summary (MCS) scores were derived. PCS and MCS scores < 50 were classified as below norm and were not interpreted as diagnostic cutoffs. Multivariable logistic regression models were used to examine the associations of habitual snoring with below-norm HRQoL after adjustment for sociodemographic characteristics, health behaviors, BMI, and chronic disease. As a sensitivity analysis, PCS and MCS were additionally analyzed as continuous outcomes using multivariable linear regression models.

**Results:**

Of the 2,312 participants (mean age 44.4 years; 63% female), 36.9% reported habitual snoring (≥3 nights/week). Habitual snorers had significantly lower scores on both the Physical Component Summary (PCS: 42.9 vs. 46.0, *P* < 0.001) and the Mental Component Summary (MCS: 49.9 vs. 50.8, *P* = 0.023) compared to non-snorers. After multivariable adjustment, habitual snoring was associated with higher odds of below-norm physical HRQoL (ORm = 1.590, 95% CI: 1.291–1.957) and mental HRQoL (ORm = 1.250, 95% CI: 1.047–1.493). In sensitivity analyses, habitual snoring remained significantly associated with lower PCS (β = −2.38, 95% CI: −3.00 to −1.75) and lower MCS (β = −1.21, 95% CI: −2.04 to −0.38).

**Conclusion:**

Habitual snoring was common among adults attending community health centers in Shanghai and was associated with lower physical and mental HRQoL, particularly physical HRQoL. These findings support greater attention to simple symptom-based screening for snoring in community care, where it may help identify individuals who may benefit from further sleep-related evaluation and health management.

## 1 Introduction

Habitual snoring is one of the most common sleep-related symptoms in adults and is increasingly recognized as more than a minor social nuisance (1–3). It reflects vibration of upper-airway structures during sleep and often indicates increased upper-airway resistance. Although it is a hallmark symptom of obstructive sleep apnea (OSA), a substantial proportion of snorers do not meet diagnostic criteria for OSA (2, 3). Population-based evidence suggests that snoring prevalence increases with age, is higher in men than in women, and is closely linked to adiposity and adverse lifestyle factors such as smoking and alcohol consumption (1, 3–6).

Beyond its acoustic and social consequences, habitual snoring has broader health implications (5, 7–9). A growing body of epidemiological evidence links snoring to cardiometabolic risk, including hypertension, impaired glucose metabolism, and type 2 diabetes (7, 9, 10). In a large population-based prospective cohort, self-reported habitual snoring was independently associated with incident hypertension, even after adjustment for major confounders, and symptom frequency showed a dose-dependent relationship with risk (9). Likewise, community-based data have shown graded associations between snoring characteristics and prediabetes or diabetes, reinforcing the view that snoring may serve as a practical marker of broader cardiometabolic vulnerability (7, 10).

Habitual snoring may also be associated with lower health-related quality of life (HRQoL), a multidimensional construct encompassing perceived physical, emotional, and social well-being (11). Several plausible pathways may help explain this association. Recurrent upper-airway obstruction and sleep fragmentation may be linked to poorer sleep continuity, reduced vitality, daytime fatigue, and functional limitation (5, 12, 13). At the same time, snoring may disturb the sleep of bed partners, strain interpersonal relationships, and increase psychological stress (1, 3, 14). Previous studies have shown that snoring and other sleep-related breathing symptoms are associated with poorer sleep quality, greater daytime anxiety, higher risk of depression and stress, and broader psychosocial burden (12–14). However, much of the literature in this area has focused on clinically diagnosed OSA, specialty sleep cohorts, or selected patient populations, rather than adults encountered in routine community-based care.

In real-world primary care and community settings, polysomnography is often unavailable, and sleep-related symptoms are more likely to be identified through brief symptom inquiry than through formal diagnostic testing (15, 16). Recent prospective evidence has highlighted the public health relevance of symptom-based identification of snoring and daytime sleepiness and has suggested that these simple self-reported symptoms may be useful screening signals in primary care (9). Yet the specific burden of self-reported habitual snoring on HRQoL remains insufficiently characterized, particularly in Chinese community-based populations.

The need to clarify this association is especially relevant in China's community health centers (CHCs), which play a central role in first-contact care and chronic disease management. Adults presenting to CHCs frequently have hypertension, diabetes, obesity, smoking exposure, and other factors that cluster with sleep-disordered breathing (17, 18). If habitual snoring is overlooked in this setting, a potentially useful marker of reduced health status may remain unrecognized.

Therefore, we conducted a cross-sectional study among adults attending community health centers in Shanghai, China, to examine the association between self-reported habitual snoring and physical and mental HRQoL.

## 2 Methods

### 2.1 Study design and setting

This cross-sectional study was conducted between October and December 2022 in Shanghai, China. To ensure geographic and socioeconomic representativeness, a multi-stage stratified sampling strategy was employed. First, three administrative districts were selected to represent distinct levels of urbanization: Xuhui (urban), Minhang (peri-urban), and Jinshan (rural). Second, seven CHCs were randomly selected within these districts. Finally, participants were recruited via convenience sampling from patients visiting these centers during the study period. The inclusion criteria were: (1) permanent residents of Shanghai aged ≥18 years; and (2) willingness and ability to provide written informed consent. Exclusion criteria included: (1) severe hearing or speech impairments that hindered communication; and (2) cognitive impairment or psychiatric conditions preventing comprehension of the survey content. It is important to note that data collection coincided with the late phase of the COVID-19 pandemic in China. During this period, localized public health measures remained in effect, a context that may have influenced population sleep patterns and psychological well-being.

### 2.2 Sample size calculation

The sample size was estimated using PASS software. Based on previous epidemiological data indicating a habitual snoring prevalence of 21.0% (19), with a precision of 3% and a confidence level of 95%, the initial calculated sample size was 745. Considering a design effect of 2.0 due to the multi-stage sampling approach, and anticipating a non-response or invalid response rate of approximately 20%, the final required sample size was determined to be at least 1,862 participants.

### 2.3 Data collection and quality assurance

Data collection was conducted using a professional electronic survey platform (Wenjuanxing, Changsha Ranxing Information Technology Co., Ltd., China) equipped with built-in quality control features, including real-time logical validation and automated response verification. The survey team consisted of qualified health researchers who received standardized training in survey methodology. To adhere to local infection prevention protocols, surveys were conducted in designated, well-ventilated rooms. Participants accessed the questionnaire via encrypted QR codes on their personal mobile devices or provided sanitized tablet devices. Standardized interviewer-assisted administration was available for participants with limited digital literacy. Prior to participation, researchers provided a standardized orientation explaining the study objectives and confidentiality measures. The platform's integrated system continuously monitored response patterns and completion times (estimated at 20–25 min). Ultimately, 2,372 participants completed the survey. After rigorous quality control, 2,312 questionnaires were deemed valid (validity rate: 97.5%), satisfying the sample size requirement.

### 2.4 Measures

#### 2.4.1 Sociodemographic and clinical characteristics

Sociodemographic data included age, sex, education level, employment status, monthly household income, marital status, and residential area. Age was categorized as < 45 and ≥45 years for descriptive and regression analyses. Health behaviors assessed included current smoking and alcohol consumption. Body Mass Index (BMI) was calculated from self-reported height and weight, and categorized according to the Working Group on Obesity in China (WGOC) criteria: normal/underweight (< 24.0 kg/m^2^), overweight (24.0–27.9 kg/m^2^), and obese (≥28.0 kg/m^2^) (20). Chronic diseases were ascertained through self-reported physician diagnoses.

#### 2.4.2 Self-reported habitual snoring

Habitual snoring was assessed using the snoring frequency item from the validated Berlin Questionnaire (16): “How often do you snore?” Response options included “Nearly every day”, “3–4 times a week”, “1–2 times a week”, “1–2 times a month”, or “Never or nearly never”. Consistent with established epidemiological conventions, participants reporting snoring “Nearly every day” or “3–4 times a week” were classified as habitual snorers; all other responses were categorized as non-habitual snorers (21).

#### 2.4.3 Health-related quality of life (HRQoL)

HRQoL was assessed using the validated Chinese version of the 12-item Short Form Health Survey (SF-12), which has demonstrated good reliability and validity in Chinese community populations (22). The SF-12 comprises 12 items covering eight domains: general health (GH), physical functioning (PF), role-physical (RP), bodily pain (BP), vitality (VT), social functioning (SF), role-emotional (RE), and mental health (MH). Following standard scoring procedures, item responses were recoded and transformed into eight domain scores ranging from 0 to 100, with higher scores indicating better health status. These domain scores were then standardized and aggregated into two norm-based summary measures: the Physical Component Summary (PCS), primarily reflecting GH, PF, RP, and BP, and the Mental Component Summary (MCS), primarily reflecting VT, SF, RE, and MH. Both summary scores were standardized using norm-based scoring to a reference mean of 50 and a standard deviation of 10, with higher scores indicating better perceived physical and mental health. For the primary logistic regression analyses, PCS < 50 and MCS < 50 were operationally classified as below-norm physical and mental HRQoL, respectively (23). These thresholds indicate scores below the norm-based reference mean and do not represent diagnostic cutoffs or validated thresholds for clinically significant impairment.

### 2.5 Statistical analysis

Data analyses were performed using SPSS version 25.0. The normality of continuous variables was assessed using the Kolmogorov-Smirnov test. Descriptive statistics were presented as frequencies and percentages for categorical variables, and as means and standard deviations (SD) for normally distributed continuous variables. Differences in PCS and MCS scores across participant characteristics were examined using independent-samples *t*-tests or one-way analysis of variance (ANOVA) for normally distributed data, and Mann-Whitney U tests or Kruskal–Wallis tests for non-normally distributed data. The associations between habitual snoring and below-norm HRQoL (PCS and MCS < 50) were evaluated through univariable and multivariable logistic regression analyses. Variables demonstrating a significant association (*P* < 0.10) in the univariable analyses, alongside clinically relevant confounders, were entered into the multivariable models. Multivariable odds ratio (ORm) and 95% confidence intervals (95% CI) were calculated to quantify these associations. All statistical tests were two-tailed, with significance defined as *P* < 0.05.

As a sensitivity analysis, the associations between self-reported habitual snoring and HRQoL were further examined by treating PCS and MCS as continuous outcomes in multivariable linear regression models. The models were adjusted for the same covariates as the main analyses. Because PCS and MCS were not normally distributed, heteroskedasticity-robust standard errors were used, and β coefficients with 95% confidence intervals (CIs) were estimated.

### 2.6 Ethical Considerations

The study protocol was reviewed and approved by the Ethics Committee of Xuhui District Center for Disease Control and Prevention (Approval No: XHLL202205). Written informed consent was obtained from all participants prior to data collection, and all data were anonymized and securely stored in accordance with the Declaration of Helsinki.

## 3 Results

### 3.1 Participant characteristics and HRQoL scores

Table 1 summarizes the sociodemographic characteristics, snoring status, and HRQoL scores of all participants. A total of 2,312 respondents completed the survey, of whom 63.0% were female. The sample was relatively evenly distributed across age groups, with 51.0% aged < 45 years. Most participants had attained a college education or higher (66.0%), were employed (66.3%), and were married (81.7%). The prevalence of self-reported habitual snoring was 36.9%. Using the prespecified norm-referenced definition, 72.4% of participants had PCS scores below 50, and 44.1% had MCS scores below 50.

**Table 1:** Associations between sample characteristic and health-related quality of life among participants (*N* = 2,312).

Variables	*N* (%)	Short Form-12 (PCS)		*t*/*Z*/*F*	*P*	Short Form-12 (MCS)		*t*/*Z*/*F* statistic	*P*
		Mean	SD			Mean	SD		
**Total**	2,312 (100.0)	44.9	7.4	–	–	50.4	9.4	–	–
Socio-demographic characteristics									
Sex				3.255	**0.001**			−3.125	**0.002**
Male	855 (37.0)	45.5	7.0			49.6	9.8		
Female	1,457 (63.0)	44.5	7.6			50.9	9.1		
Age (year)				11.081	**< 0.001**			−7.108	**< 0.001**
< 45	1,178 (51.0)	46.5	6.8			49.1	9.4		
≥45	1,134 (49.0)	43.2	7.5			51.8	9.3		
Education attainment				21.559	**< 0.001**			2.510	0.081
≤ Junior high school	315 (13.6)	43.4	7.9			49.9	10.2		
Senior high school	470 (20.4)	43.5	7.4			51.3	9.5		
≥College	1,527 (66.0)	45.6	7.1			50.3	9.2		
Employment				11.985	**< 0.001**			−5.387	**< 0.001**
Employed	1,533 (66.3)	46.1	6.8			49.7	9.3		
Unemployed	779 (33.7)	42.4	7.9			51.9	9.5		
Family monthly income (RMB)				9.898	**< 0.001**			9.962	**< 0.001**
≤ 5,000	460 (19.9)	44.4	7.4			49.0	10.1		
5,001–9,999	750 (32.4)	44.1	7.3			49.8	9.6		
10,000–19,999	693 (30.0)	45.1	7.6			51.7	8.7		
≥20,000	409 (17.7)	46.4	6.7			51.2	9.2		
Residential area				21.528	**< 0.001**			4.003	**0.018**
Urban	610 (26.4)	43.7	7.7			51.2	9.3		
Peri-urban	765 (33.1)	44.4	7.3			50.4	9.7		
Rural	937 (40.5)	46.0	7.0			49.8	9.2		
Marital status				13.994	**< 0.001**			1.261	0.081
Single	306 (13.2)	46.9	6.7			47.5	9.4		
Married	1,888 (81.7)	44.6	7.4			50.9	9.3		
Divorced/widowed	118 (5.1)	43.8	7.9			50.4	9.7		
Health status									
BMI (kg/m^2^)				4.069	**0.017**			0.305	0.737
Normal/underweight	1,454 (62.9)	45.2	7.3			50.3	9.5		
Overweight	692 (29.9)	44.3	7.6			50.7	9.4		
Obesity	166 (7.2)	44.2	7.0			50.4	9.4		
With chronic disease				16.737	**< 0.001**			−1.338	0.181
No	1,506 (65.1)	46.6	6.6			50.2	9.3		
Yes	806 (34.9)	41.5	7.5			50.8	9.5		
Health behavior									
Smoking				1.273	0.203			3.492	**< 0.001**
No	2,003 (86.6)	44.9	7.5			50.7	9.4		
Yes	309 (13.4)	44.4	6.6			48.7	9.5		
Drinking				−0.584	0.603			1.247	0.261
No	1875 (81.1)	44.8	7.6			50.5	9.5		
Yes	437 (18.9)	45.0	6.3			50.0	9.3		
Self-reported habitual snoring				9.870	**< 0.001**			2.273	**0.023**
No	1,459 (63.1)	46.0	7.0			50.8	9.3		
Yes	853 (36.9)	42.9	7.6			49.9	9.5		

PCS, physical component summary; MCS, mental component summary; SD, standard deviation. Bold values show statistical significance at *P* < 0.05.

The mean (SD) scores of the Physical Component Summary (PCS) and the Mental Component Summary (MCS) were 44.9 (7.4) and 50.4 (9.4), respectively. For physical HRQoL, PCS scores differed significantly by sex, age, educational attainment, employment status, monthly household income, residential area, marital status, BMI, chronic disease status, and habitual snoring (all *P* < 0.05), whereas no significant differences were observed by smoking or drinking status (*P* > 0.05). Specifically, males, younger participants (< 45 years), and employed individuals had higher PCS scores. Participants without chronic disease had substantially higher PCS scores than those with chronic conditions (46.6 vs. 41.5, *P* < 0.001). Habitual snorers also had lower PCS scores compared with non-snorers (42.9 vs. 46.0, *P* < 0.001).

For mental HRQoL, MCS scores differed significantly by sex, age, employment status, residential area, monthly household income, smoking status, and habitual snoring (all *P* < 0.05), but not by educational attainment, marital status, BMI, chronic disease status, or drinking status (all *P* > 0.05). Females and participants aged ≥45 years had higher MCS scores. Smokers had lower MCS scores than non-smokers (48.7 vs. 50.7, *P* < 0.001), and habitual snorers also showed lower MCS scores than non-snorers (49.9 vs. 50.8, *P* = 0.023).

### 3.2 Univariable and multivariable analysis of factors associated with below-norm HRQoL

Univariate and multivariate logistic regression results are listed in Table 2. In the univariable logistic regression analysis, self-reported habitual snoring was significantly associated with both below-norm physical HRQoL (PCS < 50; ORu = 1.883, 95% CI: 1.541–2.302) and below-norm mental HRQoL (MCS < 50; ORu = 1.198, 95% CI: 1.011–1.420).

**Table 2:** Association between self-reported habitual snoring and below-norm health-related quality of life using logistic regression analysis (*N* = 2,312).

Variables	PCS < 50	Below-norm physical HRQoL		MCS < 50	Below-norm mental HRQoL	
	*N* (%)	ORu (95% CI)	ORm (95% CI)	*N* (%)	ORu (95% CI)	ORm (95% CI)
Sex						
Male	610 (71.3)	Reference	–	414 (48.4)	Reference	Reference
Female	1,063 (73.0)	1.084 (0.898, 1.308)	–	605 (41.5)	0.756 (0.638, 0.896) ^**^	0.775 (0.649, 0.924) ^**^
Age (year)						
< 45	778 (66.0)	Reference	–	586 (49.7)	Reference	Reference
≥45	895 (78.9)	1.925 (1.597, 2.321)^**^	–	433 (38.2)	0.624 (0.529, 0.736)^**^	0.606 (0.505, 0.727)^**^
Education attainment						
≤ Junior high school	248 (78.7)	Reference	–	154 (48.9)	Reference	–
Senior high school	365 (77.7)	0.939 (0.664, 1.382)	–	194 (41.3)	0.735 (0.551, 0.979)^*^	–
≥College	1,060 (69.4)	0.613 (0.458, 0.820) ^**^	–	671 (43.9)	0.820 (0.643, 1.045)	–
Employment						
Employed	1,040 (67.8)	Reference	–	716 (46.7)	Reference	–
Unemployed	633 (81.3)	2.055 (1.667, 2.534)^**^	–	303 (38.9)	0.726 (0.609, 0.866)^**^	–
Family monthly income (RMB)						
≤ 5,000	348 (75.7)	Reference	Reference	240 (52.2)	Reference	Reference
5,001–9,999	566 (75.5)	0.990 (0.756, 1.297)	0.922 (0.698, 1.219)	359 (47.9)	0.842 (0.667, 1.062)	0.779 (0.614, 0.998)^*^
10,000–19,999	496 (71.6)	0.810 (0.619, 1.061)	0.762 (0.577, 1.006)	270 (39.0)	0.585 (0.461, 0.743)^**^	0.519 (0.406, 0.664)^**^
≥20,000	263 (64.3)	0.580 (0.432, 0.778)^**^	0.639 (0.472, 0.864)^**^	150 (36.7)	0.531 (0.405, 0.697)^**^	0.458 (0.346, 0.608)^**^
Residential area						
Urban	461 (75.6)	Reference	–	242 (39.7)	Reference	–
Peri-urban	574 (75.0)	0.971 (0.759, 1.234)	–	336 (43.9)	1.191 (0.960, 1.478)	–
Rural	638 (68.1)	0.690 (0.548, 0.868)^**^	–	441 (47.1)	1.352 (1.099, 1.663)^**^	–
Marital status						
Single	198 (64.7)	Reference	–	173 (56.5)	Reference	Reference
Married	1,385 (73.4)	1.502 (1.163, 1.939)^**^	–	792 (41.9)	0.556 (0.435, 0.709)^**^	0.710 (0.546, 0.924)^*^
Divorced/widowed	90 (76.3)	1.753 (1.080, 2.846)^*^		54 (45.8)	0.649 (0.423, 0.994)^*^	0.800 (0.509, 1.254)
BMI (kg/m^2^)						
Normal/underweight	1028(70.7)	Reference	–	640 (44.0)	Reference	–
Overweight	515 (74.4)	1.206 (0.983, 1.480)	–	298 (43.1)	0.962 (0.801, 1.155)	–
Obesity	130 (78.3)	1.496 (1.017, 2.201)^*^	–	81 (48.8)	1.212 (0.879, 1.672)	–
With chronic disease						
No	979 (65.0)	Reference	Reference	673 (44.7)	Reference	–
Yes	694 (86.1)	3.336 (2.661, 4.181)^**^	2.996 (2.381, 3.771) ^**^	346 (42.9)	0.931 (0.783, 1.106)	–
Smoking						
No	1,435 (71.6)	Reference	–	853 (42.6)	Reference	–
Yes	238 (77.0)	1.327 (1.001, 1.760)^*^	–	166 (53.7)	1.565 (1.230, 1.991)^**^	–
Drinking						
No	1,346 (71.8)	Reference	–	816 (43.5)	Reference	–
Yes	327 (74.8)	1.168 (0.921, 1.480)	–	203 (46.5)	1.126 (0.914, 1.388)	–
Self-reported habitual snoring						
No	991 (67.9)	Reference	Reference	619 (42.4)	Reference	Reference
Yes	682 (80.0)	1.883 (1.541, 2.302)^**^	1.590 (1.291, 1.957)^**^	400 (46.9)	1.198 (1.011, 1.420)^*^	1.250 (1.047, 1.493)^*^

ORu, univariable odds ratio; ORm, multivariable odds ratio; CI, confidence interval; ^*^P < 0.05; ^**^P < 0.01; Data are presented as *n* (%), with percentages calculated within each subgroup.

For below-norm physical HRQoL, older age (≥45 years; ORu = 1.925, 95% CI: 1.597–2.321), unemployment (ORu = 2.055, 95% CI: 1.667–2.534), obesity (ORu = 1.496, 95% CI: 1.017–2.201), smoking (ORu = 1.327, 95% CI: 1.001–1.760), and the presence of chronic diseases (ORu = 3.336, 95% CI: 2.661–4.181) were associated with higher odds of below-norm physical HRQoL. In contrast, higher educational attainment, higher household income, and rural residence were associated with lower odds of below-norm physical HRQoL (all *P* < 0.05).

For below-norm mental HRQoL, female sex (ORu = 0.756, 95% CI: 0.638–0.896), older age (ORu = 0.624, 95% CI: 0.529–0.736), higher household income, and being married were associated with lower odds of below-norm mental HRQoL, whereas smoking (ORu = 1.565, 95% CI: 1.230–1.991) and rural residence (ORu = 1.352, 95% CI: 1.099–1.663) were associated with higher odds of below-norm mental HRQoL (all *P* < 0.05). Educational attainment, BMI, chronic disease status, and drinking were not significantly associated with below-norm mental HRQoL.

In the multivariable logistic regression analysis, habitual snoring remained associated with both below-norm physical HRQoL (ORm = 1.590, 95% CI: 1.291–1.957) and below-norm mental HRQoL (ORm = 1.250, 95% CI: 1.047–1.493). The presence of chronic diseases also retained a strong independent association with below-norm physical HRQoL (ORm = 2.996, 95% CI: 2.381–3.771). In addition, higher household income (≥20,000 RMB) was independently associated with lower odds of below-norm physical HRQoL (ORm = 0.639, 95% CI: 0.472–0.864) and below-norm mental HRQoL (ORm = 0.458, 95% CI: 0.346–0.608). Female sex, older age, and being married were independently associated with lower odds of below-norm mental HRQoL.

**Figure 1:**
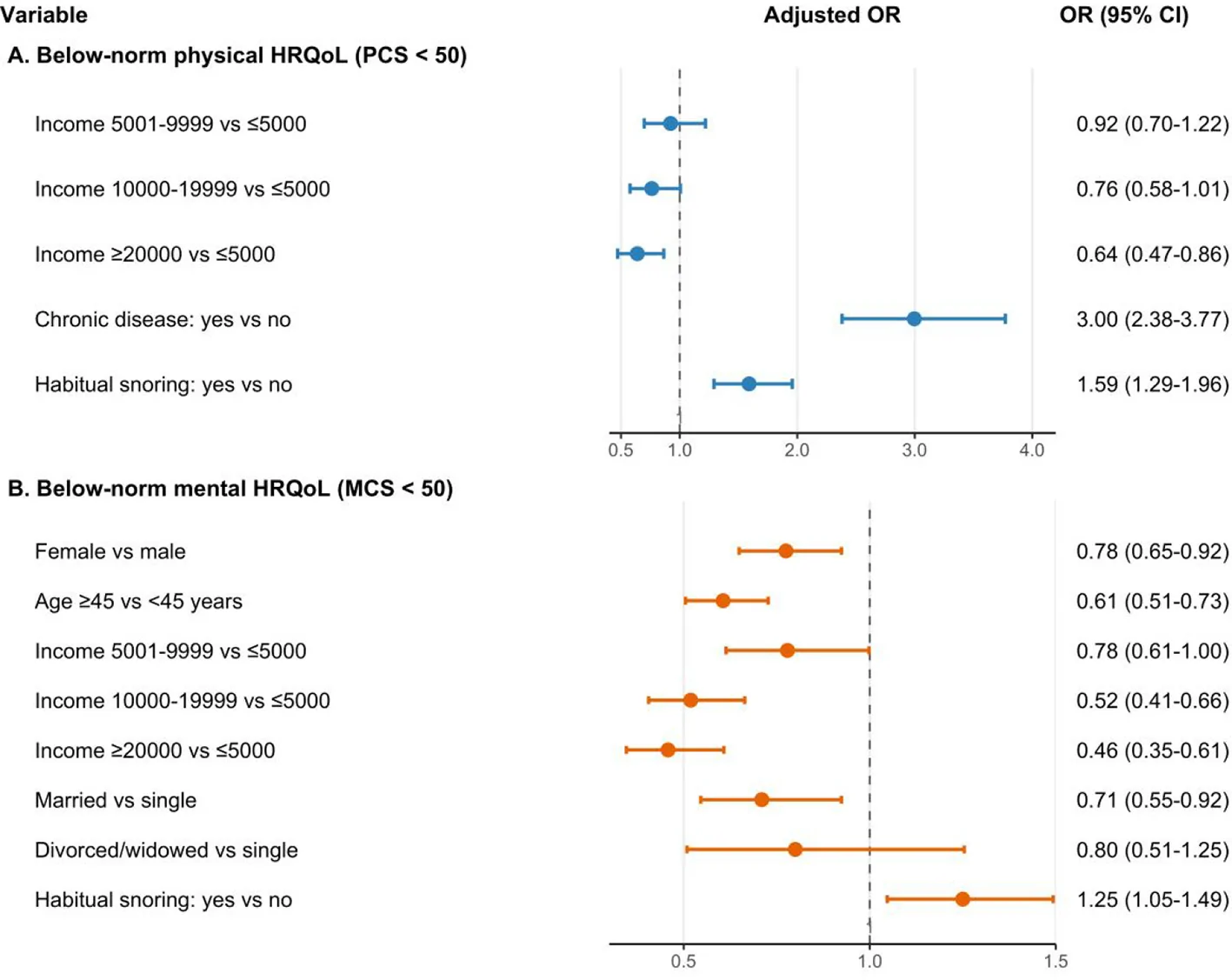
Forest plots of factors associated with below-norm physical and mental health-related quality of life. **(A)** presents adjusted ORs and 95% CIs for below-norm physical HRQoL (PCS < 50), and **(B)** presents adjusted ORs and 95% CIs for below-norm mental HRQoL (MCS < 50). The dashed vertical line indicates OR = 1.0.

Figure 1 presents the adjusted associations of factors retained in the multivariable models with below-norm HRQoL. Habitual snoring remained associated with higher odds of both below-norm physical and mental HRQoL after multivariable adjustment.

### 3.3 Sensitivity analysis

In sensitivity analyses treating HRQoL as a continuous outcome, self-reported habitual snoring remained significantly associated with lower PCS and MCS scores. After multivariable adjustment, habitual snoring was associated with a 2.38-point lower PCS score (β = −2.38, 95% CI: −3.00 to −1.75, *P* < 0.001) and a 1.21-point lower MCS score (β = −1.21, 95% CI: −2.04 to −0.38, *P* = 0.004). These findings were consistent with the main analyses using dichotomized HRQoL outcomes.

## 4 Discussion

In this cross-sectional study of 2,312 adults attending community health centers in Shanghai, 36.9% of participants reported habitual snoring. Compared with non-snorers, habitual snorers had lower PCS and MCS scores and higher odds of below-norm physical and mental HRQoL after adjustment for key sociodemographic, behavioral, and clinical factors. The association was stronger for physical than for mental HRQoL and remained robust in sensitivity analyses using continuous PCS and MCS scores. These findings suggest that habitual snoring may represent an underrecognized marker of diminished well-being in primary care populations and support greater attention to simple sleep-related symptom screening in community-based practice.

The prevalence of habitual snoring in our sample was higher than some previous estimates reported in Chinese community populations (19, 24). This difference should be interpreted cautiously. Our participants were recruited from community health centers rather than from a population-based household sample, and individuals seeking or using health services may have a higher burden of symptoms or chronic conditions than the general population. In addition, prevalence estimates of snoring vary across studies because of differences in age structure, sex composition, case definitions, and methods of ascertainment. Self-reported snoring also depends on personal awareness and, in many cases, on whether another person has observed the symptom.

The observed association between habitual snoring an d poorer HRQoL in our study is broadly consistent with previous evidence indicating that sleep-related breathing problems are associated with reduced quality of life and greater psychosocial burden (12–14). Several pathways could plausibly underlie this association, although they cannot be established in the present cross-sectional study. Habitual snoring may reflect increased upper-airway resistance and may coexist with sleep fragmentation or non-restorative sleep, which are associated with daytime fatigue, reduced vitality, and poorer physical functioning (12, 25). In some participants, self-reported snoring may also indicate undiagnosed OSA (5, 9, 26). Snoring may additionally coexist with bed-partner sleep disturbance and relationship strain, which may be associated with poorer psychosocial well-being (27).

A noteworthy finding was that the adjusted association with habitual snoring was stronger for physical than for mental HRQoL. This pattern may indicate that the observed association is more evident in somatic dimensions, such as energy, fatigue, and general health perception, than in emotional domains (12, 13), although the cross-sectional design does not permit conclusions about the underlying mechanism or temporal ordering. Mental well-being is shaped by a broad range of psychosocial factors, including work stress, family context, and wider social circumstances. Data collection (October–December 2022) also coincided with the late phase of the COVID-19 pandemic, when population-wide stressors may have influenced MCS scores and reduced the observed contrast between snorers and non-snorers (28). Nonetheless, the adjusted association between habitual snoring and MCS remained statistically significant.

Our multivariable analysis also highlighted the role of sociodemographic and clinical covariates. Consistent with prior literature, higher household income was associated with better HRQoL, whereas chronic disease was strongly associated with poorer physical HRQoL (29). Older age was independently associated with lower odds of below-norm mental HRQoL, but was not significantly associated with below-norm physical HRQoL after multivariable adjustment. The age pattern observed for mental HRQoL may be compatible with the so-called “paradox of aging,” whereby emotional well-being can remain relatively preserved or even improve despite age-related physical decline (30). This distinction further supports the value of treating HRQoL as a multidimensional outcome, with physical and mental components that may respond differently to social and clinical factors.

This study has several strengths. It was conducted in a real-world primary care setting, included a relatively large sample, and examined both physical and mental dimensions of HRQoL. We also adjusted for a range of potential confounders, including socioeconomic indicators, body mass index, health behaviors, and chronic disease. These features improve the relevance of the findings to community health practice.

Several limitations of this study warrant consideration. First, the cross-sectional design precludes the determination of causality or temporal directionality between habitual snoring and HRQoL. Second, although we employed a multi-stage strategy with random selection of CHCs to enhance geographic representativeness, participants within these centers were recruited via convenience sampling. This may introduce selection bias, as individuals seeking medical care might differ in health status from the general community population. Third, habitual snoring was assessed using a single self-reported frequency item. Some participants, particularly those without a bed partner or other witness, may have been unaware of their snoring or may have misclassified its frequency. We did not obtain bed-partner confirmation or collect related symptoms such as witnessed apneas, morning headaches, unrefreshing sleep, frequent awakenings, or excessive daytime sleepiness. We also did not conduct home sleep apnea testing or polysomnography. Consequently, exposure misclassification and residual confounding by undiagnosed OSA remain possible, and we could not distinguish primary snoring from OSA or determine whether OSA severity accounted for the observed associations. Fourth, despite adjustment for key sociodemographic and clinical covariates, residual confounding remains possible. We lacked data on objective sleep duration, specific sleep disorders (e.g., insomnia), and detailed psychological assessments (e.g., depression or anxiety scales), which are known to influence both snoring and HRQoL. Finally, the threshold of 50 was used only to identify PCS and MCS scores below the norm-based reference mean. It is not a diagnostic cutoff or a validated threshold for clinically significant impairment; therefore, the corresponding proportions should not be interpreted as prevalence estimates of a clinical disorder.

Despite these limitations, the findings have practical implications for community health services. In settings where formal sleep testing is not routinely available, a brief question about snoring frequency may help identify adults who may benefit from further sleep assessment, particularly those with chronic disease or other risk factors for sleep-disordered breathing. Complementary evidence from a recent polysomnography (PSG)-based sleep-laboratory study showed that neck circumference, waist circumference, and Epworth Sleepiness Scale scores were associated with objectively defined OSA severity, although most anthropometric correlations were weak and BMI was not significantly associated with the apnea–hypopnea index (31). Taken together, symptom reports and routinely collected clinical measures may support initial risk assessment, but they should be regarded as adjuncts rather than substitutes for objective sleep testing.

Future studies could improve on the present design in several ways. Longitudinal and broader population-based studies are needed to clarify temporal relationships and improve generalizability. Snoring should be characterized using repeated self-report, bed-partner or witness confirmation, validated screening instruments, and related symptoms such as witnessed apneas, morning headaches, unrefreshing sleep, frequent awakenings, and excessive daytime sleepiness. Incorporating home sleep apnea testing or polysomnography would allow primary snoring to be distinguished from OSA and would permit assessment of the apnea–hypopnea index, oxygen desaturation, arousal burden, and objective snoring intensity. More comprehensive measurement of sleep duration and quality, insomnia symptoms, psychological distress, medication use, and other relevant clinical factors would also permit better control of residual confounding.

## 5 Conclusion

In conclusion, self-reported habitual snoring was common among adults attending community health centers in Shanghai and remained associated, after multivariable adjustment, with lower physical and mental HRQoL, with a stronger association in the physical domain. These findings support greater attention to simple symptom-based screening for snoring in community care, where it may help identify individuals who warrant further sleep-related evaluation and health management.

## Data Availability

The datasets presented in this article are not readily available because the datasets generated and/or analyzed during the current study are not publicly available due to participant confidentiality and restrictions imposed by the ethics approval, but are available from the corresponding author on reasonable request. Requests to access the datasets should be directed to caiyong202028@hotmail.com.
