# Supplementary material for: Self-reported habitual snoring and health-related quality of life among adults attending community health centers in Shanghai, China: a cross-sectional study
Source: Front Public Health. 2026 Aug 24;14:1891156. doi: 10.3389/fpubh.2026.1891156 (PMC13547296; doi:10.3389/fpubh.2026.1891156)
Supplement: Supplementary file 1 [file Table_1.docx]

**Supplementary Table S1.** Sensitivity analyses using multivariable linear regression models with PCS and MCS as continuous outcomes

| **Outcome** | **Variable** | **β** | **95% CI** | **P value** |
| --- | --- | --- | --- | --- |
| PCS | Habitual snoring: yes vs no | -2.38 | -3.00 to -1.75 | <0.001 |
| PCS | Female vs male | -1.68 | -2.38 to -0.98 | <0.001 |
| PCS | Age ≥45 vs ≤44 years | -0.25 | -1.02 to 0.51 | 0.517 |
| PCS | Senior high school vs ≤Junior high school | -0.04 | -1.08 to 1.00 | 0.942 |
| PCS | ≥College vs ≤Junior high school | 0.21 | -0.79 to 1.21 | 0.682 |
| PCS | Married vs single | -0.55 | -1.40 to 0.31 | 0.208 |
| PCS | Divorced/widowed vs single | -0.20 | -1.73 to 1.33 | 0.800 |
| PCS | Unemployed vs employed | -1.15 | -1.96 to -0.34 | 0.005 |
| PCS | Income 5001–9999 vs ≤5000 | -0.22 | -1.07 to 0.62 | 0.607 |
| PCS | Income 10000–19999 vs ≤5000 | 0.60 | -0.29 to 1.48 | 0.188 |
| PCS | Income ≥20000 vs ≤5000 | 1.11 | 0.10 to 2.12 | 0.031 |
| PCS | Peri-urban vs urban | -0.12 | -0.87 to 0.64 | 0.763 |
| PCS | Rural vs urban | 0.81 | 0.02 to 1.60 | 0.043 |
| PCS | Underweight vs normal | -0.93 | -2.34 to 0.47 | 0.194 |
| PCS | Overweight vs normal | -0.07 | -0.74 to 0.60 | 0.834 |
| PCS | Obesity vs normal | -0.11 | -1.24 to 1.01 | 0.843 |
| PCS | Chronic disease: yes vs no | -3.64 | -4.36 to -2.93 | <0.001 |
| PCS | Drinking: yes vs no | 0.09 | -0.69 to 0.86 | 0.825 |
| PCS | Smoking: yes vs no | -0.98 | -1.87 to -0.10 | 0.030 |
| MCS | Habitual snoring: yes vs no | -1.21 | -2.04 to -0.38 | 0.004 |
| MCS | Female vs male | 0.89 | -0.05 to 1.83 | 0.064 |
| MCS | Age ≥45 vs ≤44 years | 2.44 | 1.38 to 3.49 | <0.001 |
| MCS | Senior high school vs ≤Junior high school | 1.13 | -0.31 to 2.56 | 0.125 |
| MCS | ≥College vs ≤Junior high school | 1.11 | -0.32 to 2.54 | 0.129 |
| MCS | Married vs single | 2.09 | 0.88 to 3.29 | <0.001 |
| MCS | Divorced/widowed vs single | 1.69 | -0.41 to 3.79 | 0.114 |
| MCS | Unemployed vs employed | 1.57 | 0.49 to 2.66 | 0.005 |
| MCS | Income 5001–9999 vs ≤5000 | 1.00 | -0.19 to 2.19 | 0.099 |
| MCS | Income 10000–19999 vs ≤5000 | 3.05 | 1.84 to 4.25 | <0.001 |
| MCS | Income ≥20000 vs ≤5000 | 2.72 | 1.31 to 4.13 | <0.001 |
| MCS | Peri-urban vs urban | 0.05 | -0.96 to 1.06 | 0.928 |
| MCS | Rural vs urban | -0.04 | -1.06 to 0.97 | 0.933 |
| MCS | Underweight vs normal | -2.17 | -4.27 to -0.06 | 0.043 |
| MCS | Overweight vs normal | 0.48 | -0.39 to 1.35 | 0.278 |
| MCS | Obesity vs normal | 0.89 | -0.64 to 2.43 | 0.255 |
| MCS | Chronic disease: yes vs no | -1.25 | -2.21 to -0.28 | 0.011 |
| MCS | Drinking: yes vs no | 0.79 | -0.29 to 1.88 | 0.153 |
| MCS | Smoking: yes vs no | -1.38 | -2.68 to -0.07 | 0.038 |
